# Learning from *Salicornia*: Physiological, Biochemical, and Molecular Mechanisms of Salinity Tolerance

**DOI:** 10.3390/ijms26135936

**Published:** 2025-06-20

**Authors:** Chamara L. Mendis, Rasanie E. Padmathilake, Renuka N. Attanayake, Dinum Perera

**Affiliations:** 1 Department of Bioprocess Technology, Faculty of Technology, Rajarata University of Sri Lanka, Mihintale 50300, Sri Lanka; dchamara1997@gmail.com; 2 Department of Plant Sciences, Faculty of Agriculture, Rajarata University of Sri Lanka, Puliyankulama, Anuradhapura 50008, Sri Lanka; rasaniep@agri.rjt.ac.lk; 3 Department of Plant and Molecular Biology, Faculty of Science, University of Kelaniya, Kelaniya 11300, Sri Lanka; renuka@kln.ac.lk

**Keywords:** halophytes, multi-omics, saline agriculture, salt-responsive genes, signaling pathways, transgenic plants

## Abstract

*Salicornia* species are halophytic plants that thrive in environments with moderate to high salinity. Owing to its high nutritional value and diverse bioactive constituents, *Salicornia* holds promise for applications in the food, feed, pharmaceutical, cosmetic, and bioenergy sectors. Understanding its salt tolerance mechanisms is important for developing crops suited to saline soils and water. Recent studies have revealed that *Salicornia* adapts to salinity through diverse physiological, biochemical, and molecular strategies. Despite these advances, a comprehensive synthesis of existing knowledge remains absent, hindering its effective application in crop improvement. In this review, recent advances in the understanding of *Salicornia*’s salinity tolerance are synthesized, with emphasis placed on key mechanisms: cell wall nano-mechanics, ion regulation and compartmentation, antioxidant defense, osmotic balance, phytohormonal control, signal transduction, transcriptional regulation, and the expression of salt-responsive proteins. The interactions among these mechanisms are also examined, along with their roles in conferring tolerance to additional abiotic stresses such as drought, submergence, and extreme temperatures. Finally, the potential applications of these findings in genetic engineering for improving salt tolerance in crops are discussed, along with proposed directions for future research to promote the use of halophytes in sustainable agriculture.

## 1. Introduction

Land salinization, a formidable obstacle to global agriculture, affects 20% of total cultivated lands and 33% of irrigated agricultural lands worldwide [1,2]. Improper agricultural practices, deforestation, and climate-driven factors such as rising sea levels, global warming, and tidal changes contribute to land salinization. The devastating effects of salinization are projected to intensify by 2050, leading to severe salinization of fertile agriculture land across the globe [1,3,4,5,6,7,8].

Excessive salinity hampers water and nutrient uptake by roots, causing stress and drastic reduction of overall production in most of major crop plants [9,10]. Furthermore, population growth demands a 35% to 56% increase in global food production between 2010 and 2050, exerting more pressure on degrading land resources [10].

Traditional approaches to alleviating the adverse effects of salinity on crops, such as the use of chemical amendments and conventional breeding for salt tolerant cultivars seem inefficient and time-consuming [11]. An alternative approach of utilizing halophytes (i.e., salt tolerant plants) in remediating saline soil has emerged and been effectively applied in different regions of the globe, including the Mediterranean basin, the Middle East, and African countries [12,13].

The majority of plants (98–99%) are glycophytes, which are intolerant of salinity and grow in non-saline environments [14,15], whereas halophytes are plants that can grow and complete their life cycle in highly saline environments, typically at salinity levels of 200 mM NaCl or higher [14,16,17].

Based on the eco-physiological aspects, halophytes can be distinguished mainly as obligate and facultative halophytes [18,19]. Obligate halophytes exhibit optimum growth in higher salinity (NaCl 0.1–5%) and are unable to thrive at lower salinity environments. Meanwhile, facultative halophytes have ability to grow in saline environments but they prefer low-salt or non-saline habitats. In addition, another type of halophyte has been defined: habitat-independent halophytes, which are able to tolerate saline conditions but are not native to saline habitats and prefer non-saline conditions [18,19].

Halophytes are further classified into pseudohalophytes, recretohalophytes, and euhalophytes based on their salt tolerance mechanism. Pseudohalophytes (salt-excluding halophytes) minimize transport of salt to the aerial parts by forming apoplastic barriers in the roots and through interveinal recycling of ions, thereby protecting the shoot tissues. Mangrove species commonly exhibit this adaptation [18,20]. Recretohalophytes (such as *Avicennia* spp. and *Chenopodium* spp.) protects cells by expelling excess salts via specialized structures such as salt glands and salt bladders [18]. Conversely, euhalophytes—including *Salicornia* spp. and *Suaeda* spp., both members of the family Amaranthaceae—are salt-accumulating plants that dilute absorbed salts in their succulent, water-storing tissues and compartmentalize them into vacuoles [18,21,22].

These adaptive mechanisms can be categorized into salt resistance and salt tolerance strategies [16,18]. Salt resistance mechanisms help plants minimize the harmful effects of salts by preventing their penetration into tissues, whereas salt tolerance mechanisms mitigate the adverse effects of salts once they have entered the plant [16]. Halotolerant plants serve as a precious gene pool in genetic engineering, molecular breeding, and understanding molecular and cellular mechanisms of salt tolerance and avoidance [23,24,25].

Among many such halophytes, *Salicornia* plays a significant role, with evolved intricate adaptive strategies to hamper the adverse effects of soil salinization on their growth and reproduction [16,26]. Members of the genus *Salicornia* are salt-loving annual herbaceous plants in the family Amaranthaceae [27], a group with 64 accepted species which typically thrive in saline environments [27,28]. Although seed germination and early seedling development are favored under non-saline conditions [29,30]—and these stages are particularly sensitive to salinity [31]—many well-known annual *Salicornia* species, including *Salicornia europaea* and *Salicornia brachiata*, exhibit optimal growth at around 200 mM NaCl [30,32,33]. Some *Salicornia* species are even capable of surviving at salinity levels as high as 1000 mM NaCl [32,33].

Beyond high tolerance to salinity, *Salicornia* provides numerous benefits in the food, forage, pharmaceutical, cosmetics, and biofuel industries [27,34]. Bioactive phytochemicals of *Salicornia* are renowned for various antioxidant, anti-inflammatory, antibacterial, anticancer, antidiabetic, antihypertensive, and immunomodulatory properties [27,34].

Adapted to thrive in saline environments, *Salicornia* exhibits several unique structural traits [27]. Its shallow root system efficiently absorbs water and nutrients from the upper soil layers, minimizing exposure to deeper, salt-saturated, and anoxic zones [35,36]. Additionally, its anomalous secondary thickening provides further support for survival under high salinity [28,37]. However, unlike many other halophytes, *Salicornia* species lack specialized salt-excreting structures such as salt bladders [27,28]. Instead, they rely on internal mechanisms to manage salt stress—primarily by diluting salts in their succulent, water-storing parenchyma tissues and sequestering them into vacuoles, especially within shoot endodermal tissues [27,38]. Nevertheless, *Salicornia*’s salinity tolerance is a complex, multigenic trait governed by a dynamic interplay of morphological adaptations, physiological processes, biochemical pathways, and molecular regulatory networks [20,39,40,41,42].

Many genes associated with the salt tolerance of *Salicornia* have been cloned and functionally characterized, highlighting its potential as a model halophyte for studying molecular mechanisms of salt tolerance [40,43,44,45,46]. Furthermore, extensive studies have explored its biochemical, physiological, anatomical, genomic, transcriptomic, proteomic, and metabolomic responses to salt stress [39,40,41,47,48]. However, despite such research advances, no comprehensive review has been conducted to synthesize and analyze this knowledge to highlight key findings and guide future research and industrial applications.

**Figure 1:**
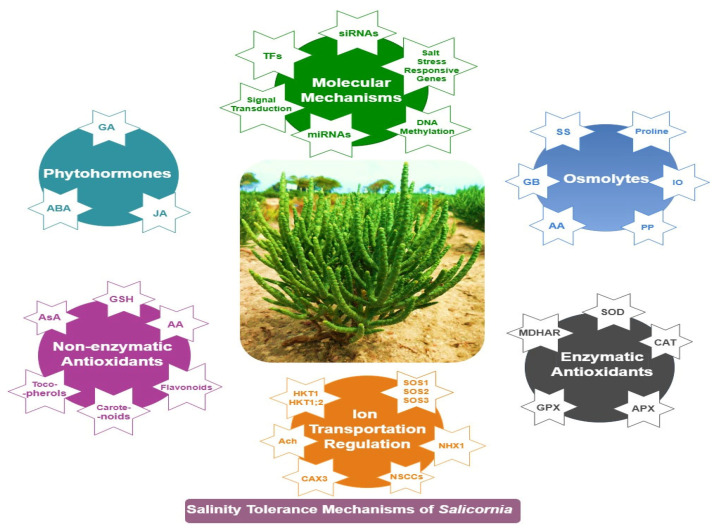
An overview of salinity tolerance mechanisms of *Salicornia*. Enzymatic antioxidants: SOD (Superoxide dismutase), CAT (Catalase), MDHAR (Monodehydroascorbate reductase), GPX (Guaiacol peroxidase). Non-enzymatic antioxidants: AsA (Ascorbic acid), GSH (reduced glutathione), AA (Amino acids), flavonoids, carotenoids, tocopherols. Phytohormones: ABA (Abscisic acid), JA (Jasmonates), GA (Gibberellins). Osmolytes: AA, PP (Polyphenols), SS (Soluble sugars), IO (Inorganic osmolytes), Proline, GB (Glycine betaine). Ion transportation regulatory elements: HKT (High affinity K^+^ transporters), NHX (Na^+^/H^+^ antiporter), NSCCs (Non-selective cation channels), SOS (Salt overly sensitive transporters), Ach (Acetylcholine), CAX (Ca^2+^/H^+^ exchanger). Molecular mechanisms: salt stress-responsive genes, TFs (Transcription factors), siRNAs (Small non-coding RNAs), miRNAs (MicroRNAs), signal transduction regulation, DNA methylation.

In light of this gap, we provide an in-depth review of *Salicornia*’s adaptive mechanisms for salinity tolerance: cell wall nano-mechanics, ion regulation and compartmentation, antioxidant defense, osmotic balance, phytohormonal control, signal transduction, transcriptional regulation, and the expression of salt-responsive proteins (Figure 1). Concurrently, we discuss interactions among these mechanisms and the multiple abiotic stress tolerance of *Salicornia*, highlighting its potential for genetic engineering applications in crop improvement. By leveraging *Salicornia*’s genetic traits, we aim to ensure sustainable agriculture in an increasingly salinized world.

## 2. Cell Wall Nano-Mechanics

Salinity stress imposes significant mechanical and osmotic challenges on plant cells, particularly due to cellular dehydration and fluctuating turgor pressure [49,50]. One critical yet often underappreciated component of a plant’s adaptive response is the nano-mechanical behavior of its cell walls. Cell wall nano-mechanics refers to the physical properties of the cell wall—such as stiffness, elasticity, and tensile strength—which are closely linked to its biochemical composition and structure [27,43,51].

**Figure 2:**
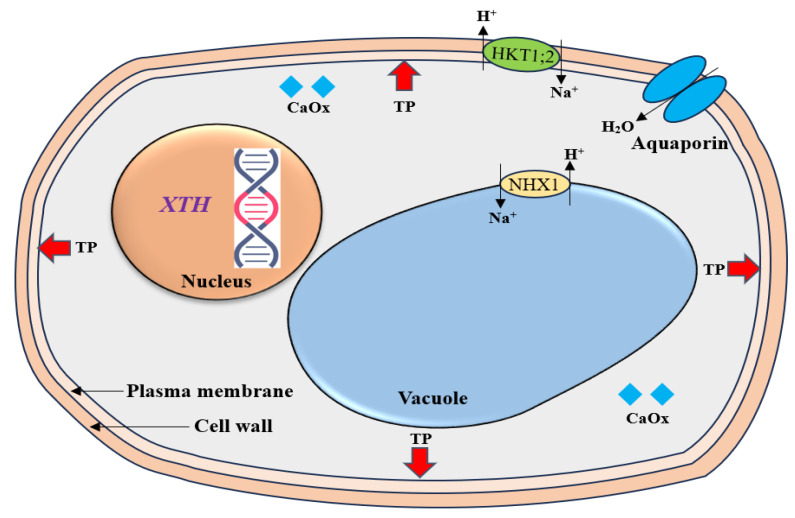
Schematic diagram representing the mechanisms of turgor pressure balancing during salt stress in *Salicornia*. High-salinity conditions cause accumulation of more Na^+^ inside cells. Succulent tissues absorb more water to dilute the Na^+^ and maintain the ionic homeostasis. Water absorbance results in increased cell volume, creating turgor pressure (TP) on the cell wall. *Salicornia* is able to soften its wall to facilitate this swelling process. Calcium oxalate (CaOx) crystal formation assists in maintaining cellular osmotic balance, accelerates photosynthesis, and provides structural support under salinity stress. *XTH* family genes in *Salicornia* are crucial for modifying the cellulose–xyloglucan composite structure of cell wall to cope with salinity stress.

Under saline conditions, excessive external salt concentrations cause osmotic water loss from cells, leading to reduced turgor pressure and cellular shrinkage [52]. If turgor cannot be maintained, key physiological functions can be impaired. Therefore, plants that can dynamically modify the mechanical properties of their cell walls—by adjusting elasticity and reinforcing structural integrity—are better equipped to tolerate salinity [43,53,54]. These modifications support osmoregulation and help stabilize turgor pressure (Figure 2) under salt-induced dehydration stress, making cell wall nano-mechanics a vital component of salinity tolerance.

Glycophytes exposed to salinity and dehydration stress typically undergo cellular water loss, leading to cell shrinkage and impaired physiological function [49,55]. In contrast, succulent halophytes increase cell volume under high salinity to store water in specialized tissues, which helps dilute salts [51,56,57]. Therefore, halophytes serve as an excellent source for understanding the genetic basis of maintaining turgor pressure in salinized environments.

Cell wall elasticity is a significant trait that facilitates the water storage capacity in tissues, allowing changes in cell size with the influx/efflux of water [43,51,53]. The effects of different salinity levels on the water-storing tissues of *Salicornia europaea* plants have been assessed through atomic force microscopy [58]. According to the stiffness values under different salt levels, it was revealed that salinity induces changes in cell wall elasticity, known as the cell turgor conversion effect. In addition to cell wall elasticity, the swelling of cells under different salt treatments was evaluated in the study, which showed a 5.4-fold swelling of cells exposed to 1000 mM NaCl compared to the control [58], suggesting a substantial increase in cell wall elasticity. These succulent tissues are crucial for maintaining ionic and osmotic homeostasis under saline conditions.

The formation and dissociation of calcium oxalate crystals serve as important adaptive mechanisms in plant responses to salinity stress [43,58]. Crystal formation helps sequester excess Ca^2+^ and oxalate ions, thereby contributing to the maintenance of ionic homeostasis and osmotic balance under high-salt conditions [58,59]. Conversely, the controlled dissociation of these crystals can release calcium ions to stabilize cellular ion levels and provide carbon dioxide to support photosynthesis as needed [58,60]. This dynamic regulation allows plants to respond flexibly to changing physiological demands under saline environments. Scanning electron microscope X-ray microanalysis of *Salicornia europaea* water-storing parenchyma cells revealed that plants treated with 1000 mM NaCl synthesized larger calcium oxalate crystals compared to those under lower or no salt treatment [58]. The formation of calcium oxalate crystals is believed to aid plants in thriving under salinity stress by enhancing cellular calcium regulation [43,60], supporting photosynthesis in a carbon dioxide-limited environment when stomata are closed to prevent water loss and contributing to cellular mechanical strength [60].

Xyloglucan, a hemicellulosic polysaccharide, contributes 20–25% of the primary cell wall dry weight in most of plants and is responsible for maintaining the integrity and extensibility of the cell wall [61,62]. The xyloglucan endotransglucosylase/hydrolase (*XTH)* family genes are crucial in remodeling cell wall architecture by cleaving and rejoining xyloglucan [61,63]. Transcriptome-wide characterization of the *XTH* multigene family of *Salicornia europaea* under salinity and drought stress discovered 35 non-redundant potential SeXTH proteins. The expression patterns of *SeXTH* genes implied their involvement in salinity tolerance, emphasizing their significance in salt stress-responsive remodeling of cell wall architecture [47].

Complementing the role of XTH genes, *Salicornia*’s capability for salinity tolerance through cell wall remodeling is further supported by studies involving laccase genes, specifically *SeLAC1* and *SeLAC2*, derived from *Salicornia europaea* [64]. Overexpressing these genes in *Arabidopsis thaliana* resulted in significant structural enhancements, including increased secondary wall thickness, elevated lignin content, and a greater number of xylem vessels [64]. Additionally, introducing the xyloglucan-related *SeXTH2* gene along with the expansin gene, *SeEXPB*, further amplified these structural changes by enlarging cells and leaves and modifying hemicellulose and pectin composition. Together, these modifications resulted in improved cell wall parameters and significantly enhanced the overall salinity tolerance of transgenic *Arabidopsis* plants [64]. The *SeXTH2* gene also demonstrated substantial efficacy in enhancing grain yield and salinity tolerance in transgenic rice, highlighting its considerable potential in genetic engineering applications aimed at salt tolerance improvement [64].

Expanding beyond xyloglucan-associated genes, thaumatin-like proteins (TLPs) constitute another important class of molecules involved in cell wall remodeling and contribute significantly to plant tolerance against both biotic and abiotic stresses, including drought, salinity, heat, and cold [65,66,67]. In *Salicornia europaea*, the *SeNN24* gene encoding a TPL-like protein conferred notable salinity tolerance in yeast, supporting growth at NaCl concentrations up to 1.3 M through modifications in cell wall composition [68]. Moreover, this gene’s salinity-tolerance-promoting effect was confirmed in transgenic tobacco plants, with significant tolerance improvements at salinity levels up to 400 mM NaCl [69]. Further research into *SeNN24* is warranted to deepen the understanding of its molecular and physiological mechanisms.

Furthermore, the structural integrity and resilience provided by cuticular waxes are vital for plant adaptation to drought and salinity stresses [57,70,71]. *Salicornia europaea* responds to increased salinity by enhancing cuticle thickness and overall wax deposition. Correspondingly, the upregulation of genes involved in cuticular wax biosynthesis, notably *SeFAR1*, *SeFAR2*, and *SeFAR3*, confirms their active role in facilitating salinity stress responses [72]. Future research and cross-species validations will be essential to elucidate the detailed biochemical pathways and physiological relevance of cuticular wax under salinity stress conditions.

Altogether, these findings suggest a model where *Salicornia* utilize both biomechanical and biochemical signaling to regulate cell wall elasticity, osmotic adjustment, and stress-responsive gene expression. Understanding these mechanisms, including the crosstalk between wall-loosening enzymes, cell turgor sensors, and ionic regulators, provides valuable insights into bioengineering strategies for enhancing salt tolerance in glycophytic crops.

## 3. Ion Transport Regulation and Compartmentalization

Exposure to excessive salt is a major challenge for plants growing in saline conditions. Na^+^, the most prominent cation in saline soil solution, causes osmotic stress, ion toxicity, and nutrient deficiency in plants [49,50]. Accumulation of excessive Na^+^ in cytoplasm creates ion imbalances and damage to the plasma membrane integrity, and Na^+^ competes with K^+^ for binding sites on vital cellular enzymes, inhibiting the activity of more than 50 enzymes and causing metabolic toxicity [49,73,74]. In addition, K^+^ plays an irreplaceable role in protein synthesis, solute transport, and maintenance of ionic homeostasis by neutralizing negative charges [73]. Insufficient availability of K^+^ under saline conditions hinders these biochemical and physiological functions [73,74,75].

To address these ionic stresses, *Salicornia* employs multiple strategies that are crucial for its survival under salinity stress [27,44,58,76,77]. For instance, its parenchyma cells accumulate water, diluting internal salt concentrations and maintaining turgor pressure—a mechanism termed succulence, especially pronounced in euhalophytes [19,78]. When cytosolic Na^+^ concentrations rise further, *Salicornia* effectively sequesters excess Na^+^ into the central vacuole, protecting the cytoplasm from toxicity [44,58,79]. Although glycophytes like *Arabidopsis* also compartmentalize ions, *Salicornia* achieves this more efficiently via specialized transporters and biomolecules [38,80,81,82].

Highlighting these adaptive strategies, a comparative study between a glycophyte, *Spinacia oleracea*, and *Salicornia dolichostachya* showed that at 200 mM NaCl, spinach required induction of tonoplast proton pumps and Na^+^/H^+^ antiporters, while *Salicornia dolichostachya* maintained high constitutive activity of these components without additional upregulation [83]. This intrinsic capability allowed *Salicornia* to sustain robust growth under saline conditions detrimental to spinach. These findings demonstrate the efficiency of tissue dilution and constitutive ion compartmentalization (predominantly into vacuoles of shoot endodermis tissues) of *Salicornia* and underscore its value as a model system for studying salt tolerance mechanisms [83].

Given the significance of ion compartmentalization in *Salicornia*’s salinity tolerance, it is essential to understand the biochemical pathways involved in ion transport and Na^+^ sequestration. Central to this resilience is the salt overly sensitive (SOS) signaling pathway, composed of SOS1, SOS2, and SOS3 proteins that are crucial in maintaining cellular ionic homeostasis and salt tolerance by cellular signaling under salt stress [84,85,86].

**Figure 3:**
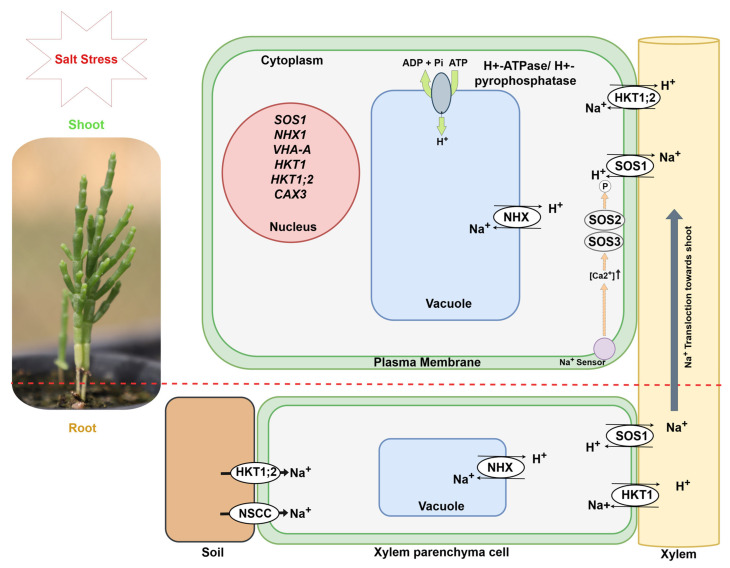
Schematic representation of plasma and tonoplast transmembrane transporters, channels, and pumps mediating Na^+^ and K^+^ homeostasis in *Salicornia* under salt stress. Na^+^ enters the cell via non-selective cation channels (NSCCs) and HKT1;2 transporters. Meanwhile, HKT1 transporters enhance the K^+^ uptake to maintain K^+^/Na^+^ homeostasis. At the xylem parenchyma cells, SOS1 loads Na^+^ into xylem sap. Tonoplast-localized NHX exchangers in shoot tissues facilitate the storage of Na^+^ in large central vacuoles. *SOS1*, *NHX1*, *VHA-A*, *HKT1*, *HKT1;2*, and *CAX3* are characterized genes from different *Salicornia* species, crucial for the ionic homeostasis.

SOS1, a Na^+^/H^+^ antiporter located in the plasma membrane, regulates Na^+^ efflux into the apoplast [31,76,87]. First identified in *Arabidopsis thaliana*, SOS1 has since been characterized in halophytic species including *Salicornia brachiata* and *Salicornia dolichostachya* [76,88]. The activity of SOS1 is modulated by SOS2 and SOS3: salt-induced calcium (Ca^2+^) signals activate the protein kinase SOS3, which then forms a complex with the serine/threonine protein kinase SOS2. This SOS2–SOS3 complex activates SOS1, thereby enhancing the plant’s ability to expel excess sodium and maintain ion homeostasis under salt stress (Figure 3) [31,87]. In addition to SOS3, plants also utilize SOS3-like calcium-binding protein 8 (SCaBP8), which functions similarly by sensing Ca^2+^ signals, binding to SOS2, and forming the SOS2–SCaBP8 complex that activates SOS1. While SOS3 activity is primarily observed in root tissues, SCaBP8 is predominantly active in shoots, as demonstrated in *Arabidopsis* and other model species [86,87].

The functional roles of SOS pathway genes in *Salicornia* have been further validated through heterologous expression in model and crop plants [88,89]. For instance, transgenic tobacco overexpressing the *SbSOS1* gene from *Salicornia brachiata* exhibited significantly greater salt tolerance than wild-type (WT) plants [88]. These transgenic lines showed improved growth, higher relative water content, better membrane stability, increased chlorophyll content, and a more favorable K^+^/Na^+^ ratio. Additionally, reductions in reactive oxygen species (ROS) accumulation and electrolyte leakage contributed to enhanced stress tolerance. Interestingly, the *SbSOS1* promoter also demonstrated the ability to regulate multiple cis-acting elements under salt stress, suggesting that its role extends beyond ion transport to broader regulatory functions that influence K^+^/Na^+^ balance across various tissues [89].

Adding to these findings, a novel insight into *Salicornia*’s adaptive strategies was provided by a proteomic study on *Salicornia bigelovii* under salinity stress. Although SOS1 is typically localized to the plasma membrane, subcellular membrane proteomics revealed that a homolog, SbiSOS1, was localized to the tonoplast (vacuolar membrane). This neo-localization enabled more efficient Na^+^ sequestration into vacuoles, thereby reducing cytosolic toxicity and enhancing salinity tolerance [82]. This finding highlights how *Salicornia* utilizes proteomic-level adaptations to withstand extreme environments. It also underscores the importance of investigating stress responses across different molecular levels to fully understand the plant’s adaptive capabilities.

Complementing SOS pathway functions, sodium/proton antiporter (NHX) proteins localized in the vacuolar membrane facilitate the sequestration of excess Na^+^ into the vacuole, as shown in Figure 3 [90,91,92], preventing its accumulation in the cytosol and enabling cells to maintain Na^+^/K^+^ homeostasis [91,92,93]. The *SbNHX1* gene demonstrated enhanced expression level when *Salicornia brachiata* was exposed to salinity stress [91]. Furthermore, tobacco plants transformed with *SbNHX1* exhibited improved salt tolerance compared to the WT tobacco [91].

Effective vacuolar sequestration depends on proton gradients established by two vacuolar proton pumps: H^+^-ATPase (V-ATPase) and pyrophosphatase (V-PPase) [42,43,44]. The role of the *SeVHA-A* gene, which encodes the H^+^-ATPase subunit in *Salicornia europaea*, has been investigated through RNA interference (RNAi)-directed downregulation in suspension-cultured cells of *Salicornia europaea* [44]. The *SeVHA*-*A* RNAi cells demonstrated lower vacuolar Na^+^ sequestration and reduced cell viability under different salinity stresses. Knockdown of *SeVHA-A* declined both V-ATPase and V-PPase activity in RNAi cells, providing insights into combined activity of both enzymes and highlighting the significance of *SeVHA-A* in salinity tolerance in *Salicornia europaea* [44,94].

In addition to vacuolar sequestration, high-affinity K^+^ transporters (HKTs) play a critical role in the long-distance transport of Na^+^ and K^+^ in plants. Two sub-classes of HKT1-type transporters have been recognized based on protein structure and ion selectivity [95,96]. Class I HKTs preferentially conduct Na^+^ over K^+^, while class II HKT transporters are Na^+^/K^+^ co-transporters, which can select Na^+^ and/or K^+^ conductance according to the requirement [97,98,99]. *SbHKT1*, encoding HKT transporters in *Salicornia bigelovi*, has been transferred and overexpressed in transgenic cotton, resulting in significantly higher biomass accumulation, germination rate, and K^+^/Na^+^ ratio in leaves, stems, and roots under salt stress compared to WT plants [95]. Additionally, a novel HKT gene isolated from *Salicornia europaea*, *SeHKT1;2*, facilitated Na^+^ uptake. Similarly, *SeHKT1;2* from *Salicornia europaea* dynamically regulated Na^+^ uptake, enhancing efficient ion management between roots and shoots during salinity stress. Under high-salinity conditions, *SeHKT1;2* was downregulated in roots and upregulated in shoots, a regulatory pattern that may reduce Na^+^ retrieval from the xylem, thereby facilitating Na^+^ transport into the shoots [96]. This spatial regulation suggests that *SeHKT1;2* plays a critical and possibly irreplaceable role in *Salicornia*’s salt tolerance capacity.

Moreover, in *Salicornia*, an acetylcholine (ACh)-mediated system has been proposed as a potential mechanism for channel-based ion transport, supported by the upregulated expression of the AChE gene in *Salicornia europaea* under salinity stress [100]. However, further studies are needed to validate the functional role of this pathway in salinity tolerance of *Salicornia*.

Salt-induced calcium (Ca^2+^) signaling, which activates the SOS2–SOS3 complex, represents just one aspect of the broader role that calcium plays in salinity stress responses [101,102]. Beyond its involvement in SOS pathway activation, cytosolic Ca^2+^ serves as a central second messenger in plant cells, mediating the perception and transduction of various abiotic stress signals [31,59,101,102]. It regulates gene expression and activates a wide range of stress-responsive proteins to maintain cellular homeostasis under high-salinity conditions [31,102,103].

Among the key regulators of intracellular calcium levels are Ca^2+^/H^+^ exchangers (CAXs), membrane transporters that sequester Ca^2+^ into vacuoles to prevent cytotoxic accumulation [31,102]. In *Salicornia europaea*, the *SeCAX3* gene—encoding a CAX transporter—has been cloned and functionally expressed in yeast, where it conferred enhanced salt tolerance. This finding highlights the contribution of multiple calcium-regulatory components to *Salicornia*’s robust salinity adaptation [52].

Collectively, Salicornia’s ion transport mechanisms operate as an interconnected network, integrating membrane potential dynamics, cytosolic ion concentrations, and gene expression. Future research employing advanced techniques, including real-time ion flux imaging and promoter-reporter assays, will further elucidate these intricate regulatory interactions [28,44,88,95].

## 4. Antioxidant Defense

### 4.1. Reactive Chemical Species and Their Crosstalk

Under salinity stress, plants experience an overproduction of reactive chemical species [104,105,106]. These species—including reactive oxygen species (ROS), reactive nitrogen species (RNS), reactive carbonyl species (RCS), and reactive sulfur species (RSS)—play dual roles in plant cells [42,107,108,109]. On one hand, they regulate growth, development, and metabolic activities through intricate signaling pathways [104,105,106,110,111,112]. On the other, their excessive accumulation, especially under biotic and abiotic stress, disrupts redox homeostasis, triggering oxidative stress and causing cellular damage [113,114]. To counteract this, plants inhabiting extreme environments have evolved adaptive strategies to manage these reactive molecules effectively [113,114,115,116,117]. Among these, ROS are the most extensively studied [107,115,118]. They are naturally produced as by-products of various cellular metabolic reactions and are involved in cellular events including maintaining normal plant growth, gene activation, long-distance signaling, and epigenetic changes [107,115,119] ROS include both free radicals—such as superoxide radical (O_2_^•−^), hydroxyl radical (•OH), alkoxyl radical (RO•), and peroxyl radical (ROO•)—and non-radical molecules, including singlet oxygen (^1^O_2_) and hydrogen peroxide (H_2_O_2_) [116,120].

Salinity stress induces the excessive accumulation of ROS, which act as primary agents of oxidative stress by reacting with cellular biomolecules and disrupting metabolism through lipid peroxidation and the denaturation of proteins and nucleic acids [107,116,120,121,122,123]. Chloroplasts, mitochondria, apoplast, and peroxisomes act as the major ROS generation sites in plants under salt stress [124,125,126,127,128]. Among ROS, ^1^O_2_ oxidizes proteins, lipids, and DNA by targeting their various residues, whereas $O_{2}^{•-}$ often reacts with iron centers in proteins and unsaturated lipids. H_2_O_2_ produces •HO by reacting with protein residues, and resulting •HO expeditiously reacts with all biomolecules [129,130]. The increased ROS level under salinity causes disfunction of the membrane activities and leakage of ions and other metabolites from the cell. ROS attack on some amino acids (AA) and DNA may be irreversible, which causes lethal effects to cells due to failure in completing crucial cellular processes [116,121,130,131].

While ROS are well characterized, other reactive species like RNS, RCS, and RSS are gaining attention for their roles under stress conditions [116,132,133,134]. RNS, particularly nitric oxide (NO) and its derivatives, also exhibit dual functionality—acting as signaling molecules or as sources of oxidative damage, depending on their concentrations and exposure duration [123,132]. Interestingly, NO’s role under salinity varies among plant species and developmental stages [133,134,135,136,137,138]. For instance, exogenous application of sodium nitroprusside (a NO donor) significantly improved salinity tolerance in *Salicornia persica* under 40 dS/m NaCl [139]. This improvement was associated with enhanced antioxidant activity, elevated proline and chlorophyll content, reduced malondialdehyde levels, and improved seed yield and oil content [139], indicating NO’s potential as a regulatory molecule in stress resilience.

RCS, which includes the α,β-unsaturated aldehydes and ketones produced from lipid peroxides, can induce antioxidant systems at low levels [118,140], but they are highly reactive and can cause severe cellular damage under high concentrations [118,140,141]. Many tau-class glutathione S-transferase isoenzymes in *Arabidopsis thaliana* have been shown to scavenge acrolein [140], a major reactive carbonyl species (RCS) in plants. A tau-class glutathione S-transferase gene from *Salicornia brachiata* has conferred salinity tolerance in transgenic tobacco, suggesting that this isoenzyme may also function as an efficient acrolein scavenger [142]. Future studies could focus on identifying similar valuable scavenging molecules from *Salicornia*.

Although classified by their reactive core elements, ROS, RNS, RCS, and RSS interact extensively, forming a complex signaling and damage network under abiotic stress [104,105,143]. For example, ROS overproduction can stimulate NO biosynthesis [17,144], while detoxification of H_2_O_2_ may suppress NO levels [16,144]. Moreover, $O_{2}^{•-}$ can react with NO to form highly toxic species like •OH and peroxynitrite (ONOO^−^), which inflict severe damage on biomolecules [144,145]. RCS can also promote ROS accumulation by modulating antioxidant enzyme activity [145,146]. RSS—formed through reactions between ROS and sulfur-containing molecules—are considered second-generation reactive species and play interconnected roles in stress responses [105,143].

Collectively, these interconnections demonstrate that reactive chemical species do not act in isolation but instead function as an integrated network in plant stress physiology. A deeper understanding of these interactions in *Salicornia*, beyond ROS alone, will provide valuable insights into redox regulation and adaptive responses to salinity.

### 4.2. Enzymatic Antioxidants

To maintain redox homeostasis under salinity stress, plants have evolved a sophisticated antioxidant defense system that detoxifies ROS, RNS, and other reactive species. This system includes a network of enzymatic antioxidants such as superoxide dismutase (SOD), catalase (CAT), ascorbate peroxidase (APX), guaiacol peroxidase (GPX), glutathione S-transferase (GST), monodehydroascorbate reductase (MDHAR), dehydroascorbate reductase, and non-enzymatic antioxidants such as ascorbic acid (AsA), reduced glutathione (GSH), α-tocopherol, carotenoids, flavonoids, and proline [129,139,147,148,149].

SOD represents the first line of enzymatic defense by catalyzing the dismutation of superoxide radicals ($O_{2}^{•-}$) into molecular oxygen (O_2_) and hydrogen peroxide (H_2_O_2_) [148]. This reaction is crucial not only for minimizing the formation of hydroxyl radicals (•OH) through the Fenton reaction [150], but also for indirectly regulating RNS levels by controlling ROS accumulation [148,151]. SOD can be classified into isozymes based on the type of prosthetic metal, namely Fe-SOD, CuZn-SOD, and Mn-SOD, which are localized in different organelles, such as mitochondria, chloroplasts, apoplasts, and peroxisomes [128,151]. Studies have reported the triggered SOD activity of several *Salicornia* species, including *Salicornia brachiata*, *Salicornia persica*, and *Salicornia europaea* seedlings, under salinity stress [48,139,149]. Further examination of SOD isoforms has revealed variations among isoforms of mitochondrial Mn-SOD, chloroplast Fe-SOD, and cytosolic Cu/Zn-SOD, suggesting the richness of SOD antioxidants in *Salicornia europaea* and *salicornia persica* [152].

While SOD neutralizes superoxide radicals, H_2_O_2_ generated from this reaction must also be detoxified [128,148]. Catalase (CAT) performs this role by converting H_2_O_2_ into water and oxygen with remarkable efficiency (up to 6 × 10⁶ molecules per minute) [153,154,155]. This enzyme is encoded by a multigene family in plants, resulting in various isoforms in plant systems, indicating its versatile role [153,154,155]. The evaluation of antioxidant activity in *Salicornia persica* and *Salicornia europaea* exposed to varying NaCl concentrations demonstrated a gradual increase in CAT activity of both species, suggesting its responsive role in enhancing salinity tolerance [152].

Another critical enzymatic system is the ascorbate–glutathione (AsA–GSH) cycle, where ascorbate peroxidase (APX) reduces H_2_O_2_ to water using ascorbate as a specific electron donor [156,157,158]. Plants are equipped with different APX isoforms in various cellular components, including stomatal cells, chloroplasts, mitochondria, peroxisomes/glyoxysomes, and cytosol, each playing a role in keeping cellular homeostasis under stress [156,157,159]. A peroxisomal ascorbate peroxidase-encoding gene in *Salicornia brachiata*, *SbpAPX* has been characterized and functionally validated by transfer into tobacco. Those transgenic tobacco plants conferred salinity stress tolerance by directly quenching H_2_O_2_, exhibiting maximum expression level at 500 mM of NaCl, and modulating overall growth and development [160]. Northern blot analysis further confirmed that *SbpAPX* transcripts were upregulated not only under salt stress but also in response to cold, abscisic acid (ABA), and salicylic acid treatments—indicating a broad-spectrum role in abiotic stress adaptation [160]. These findings suggest that *Salicornia* holds significant potential as a genetic reservoir for improving both salt and multi-stress tolerance in crops. Future studies, including genome-wide identification and functional characterization of the APX gene family in *Salicornia*, could provide deeper insights into their diverse roles in stress responses.

GPX is another crucial enzyme involved in the active elimination of H_2_O_2_ [152,161]. It functions across various subcellular compartments—including the mitochondria, cytosol, peroxisomes, and even the apoplast—both during normal metabolism and in response to stress conditions. In addition, it plays an important role in lignin synthesis, contributing to cell wall strengthening [161]. GPX activity in both *Salicornia persica* and *Salicornia europaea* seedlings increased gradually and reached a maximum at 340 mM of NaCl when treated with different concentrations for 21 days, indicating its responsiveness for different salinity levels. In addition, the activity of different GPX isoforms in both species varied across salinity levels [152], highlighting the functional diversity of enzymatic antioxidants that enables plants to neutralize various toxic compounds and enhance survival under abiotic stress.

GSTs are ubiquitous and multifunctional enzymes that catalyze a broad range of detoxification reactions [142,162,163,164]. They achieve this by binding to electrophilic and hydrophobic toxic molecules and converting them into less harmful glutathione-conjugated derivatives [165,166,167,168,169]. The GST gene family consists of 25 to 60 genes in plants belonging to six classes: phi, tau, zeta, theta, lambda, and dehydroascorbate reductase. These enzymes play a crucial role in protecting plants from different stress conditions such as salinity, drought, cold, and herbicide-toxicity [170,171]. In *Salicornia brachiata*, a GST gene in the *tau* class, *SbGSTU*, was upregulated under salt stress. Moreover, it enhanced seed germination and growth of transgenic tobacco overexpressing the *SbGSTU* gene under salt stress by quenching secondary noxious by-products [142]. Functional and molecular characterization of an *SbGSTU* gene promoter revealed the presence of several abiotic and biotic stress-responsive motifs, indicating the regulation of the expression of the *SbGSTU* by abscisic acid mediated signaling pathway under salinity stress. Quantitative GUS (β-glucuronidase) activity assay revealed the efficient expression of the reporter protein under salinity stress, implying the potential of *SbGSTU* expression to modulate salinity tolerance in transgenic tobacco [162].

Taken together, the enzymatic antioxidant system in *Salicornia* is diverse, robust, and highly responsive to salinity. It comprises spatially distributed isoforms with overlapping yet distinct roles, enabling efficient detoxification of a broad spectrum of reactive chemical species. This intricate enzymatic network underlies *Salicornia*’s remarkable ability to withstand extreme saline environments and offers valuable molecular tools for improving abiotic stress tolerance in other crop species.

### 4.3. Non-Enzymatic Antioxidants

In addition to the enzymatic antioxidants, plants are equipped with a non-enzymatic scavenging system that consists of low-molecular-weight antioxidants such as AsA, GSH, tocopherols, carotenoids, phenolics, flavonoids, and AA (such as proline) [149,163,172,173]. Many of these antioxidants are multifunctional, performing various biological roles beyond oxidative stress mitigation. For instance, flavonoids act as natural insecticides that selectively deter pests without harming beneficial insects [174]. Ascorbic acid priming has been shown to enhance seed germination and early seedling development in several crop species [175], and AsA has also demonstrated effectiveness in controlling certain plant diseases [176]. Non-enzymatic antioxidants, in particular, are able to counteract the negative effects of ROS by directly detoxifying them [17,177]. They do so by donating electrons to stabilize the free-radical ROS molecules, neutralizing their reactivity, and preventing further chain reactions within the cell [177,178].

AsA is considered the first line of defense against ROS attack due to its substantial availability in the apoplast [175,179]. It acts as an electron donor in many important cellular biochemical reactions, such as the ascorbate-GSH pathway, and protects cell membranes by supporting phospholipid regeneration via directly detoxifying H_2_O_2_, •HO the and $O_{2}^{•-}$. It also facilitates the production of α-tocopherol from tocopheroxyl free radicals generated through metabolism or stimulated by numerous stresses, thereby protecting PSII from photo-oxidation [156,157,158,179]. Ascorbate, the reduced form of AsA, is oxidized to form DHA in the H_2_O_2_ scavenging process by ascorbate. The ascorbate/DHA ratio in *Salicornia brachiata* exhibited a higher rate at low salinity (200 mM NaCl) and lower rates at high salinity levels (400 and 600 mM NaCl), which coincides with the ascorbate oxidation under high salinity to quench ROS [149].

GSH is distributed in almost all subcellular compartments of plants and is engaged in many crucial cellular processes due to its high reduction potential [163,173]. It directly detoxifies H_2_O_2_, •HO, and $O_{2}^{•-}$, forming by-products such as GSSG, which assists in the regeneration of ascorbate [163,173]. The total glutathione content (GSH + GSSG) and GSH/GSSG ratio in *Salicornia brachiata* have been significantly enhanced under salinity stress, implying its higher responsiveness for the salinity stress [149].

Tocopherols, are group of soluble phenolic compounds that play a crucial role in the protection of cell membranes from oxidative stress [172,180,181]. Among the tocopherol isomers, α-tocopherol exhibits the most robust antioxidant properties, with the ability to quench up to 120 molecules of ^1^O_2_ per molecule and regulate ^1^O_2_ level by minimizing the photo-oxidative damage [147,180,181]. The activity of tocopherols (α, γ, and δ) in *Salicornia bigelovii* seed oil has been studied for their nutritional significance as antioxidant agents [182]. Lipophilic profile analysis of different organs of *Salicornia perennis* under salt stress regimes highlighted the role of tocopherols as efficient ROS scavengers [183].

Carotenoids are pigment molecules that absorb light in photosynthesis and are the most abundant lipid-soluble antioxidants [172,184,185]. They are the most effective scavenger of the ROO• that provide defense against lipoproteins and cell membranes from lipid peroxidation, with lycopene and carotene being the most prominent members [184,186]. Carotenoids exhibit antioxidant activity through four primary mechanisms: reacting with peroxidation products to end chain reactions, detoxifying ^1^O_2_, preventing ^1^O_2_ generation by interacting with chlorophyll, and dissipating energy in the xanthophyll cycle [129,187]. Increased contents of carotenoids in two genotypes of *Salicornia neei* under salinity stress implied their role in imparting salt tolerance [187]. Phytoene synthase, a key enzyme in the carotenoid biosynthetic pathway, encoded by the gene *SePSY*, cloned from *Salicornia europaea*, improved salt tolerance in transgenic *Arabidopsis thaliana* compared to the WT, resulting in higher growth performances, enhanced SOD and POD activities, and lower contents of H_2_O_2_ and malondialdehyde (MDA), a product of lipid peroxidation [63,188]. These results highlight the potential role of carotenoids in strengthening the plant’s antioxidant defense system under salinity stress.

Flavonoids, a family of polyphenolic compounds, are well-known pigments in flowers and fruits [189,190]. They encompass several structural classes, including flavanols such as quercetin, which counteracts the oxidative degradation of DNA caused by H_2_O_2_, •HO, and $O_{2}^{•-}$, anthocyanins which protect cells from fatty acid oxidation, as well as flavones and isoflavones [189,191]. Moreover, flavonoids can be considered a secondary ROS-neutralizing system in plants because they act as barriers to highly energetic wavelengths that reach ROS-producing cells [192]. *Salicornia neei* exhibited enhanced antioxidant capacity with high total free flavonoids and quercetin when irrigated with saline shrimp farm effluent [187]. Similarly, a metabolomic analysis of *Salicornia europaea* under salinity stress revealed tissue-specific accumulation of various flavonoid compounds [193], indicating their active role in mitigating the adverse effects of salinity.

Proline, an α-amino acid derivative, was first identified as an accumulating compound in *Lolium perenne* (ryegrass) under drought stress [194,195,196]. It plays a key role in mitigating oxidative stress by scavenging reactive oxygen species such as hydroxyl radicals (•OH) and singlet oxygen (^1^O_2_) [194,197]. Moreover, proline boosts the antioxidant system indirectly by enhancing the activity of some of enzymatic antioxidants such as CAT, SOD, and APX [177,197,198]. The significant role of proline for salt tolerance of *Salicornia* has been highlighted in many studies [46,139,199,200,201]. For instance, proline demonstrated significant accumulation in *Salicornia persica* with salinity treatments beyond 400 mM NaCl, emphasizing its contribution for mitigation the stress at higher salinity levels [199].

In summary, these non-enzymatic antioxidants form a highly coordinated and spatially distributed network that complements enzymatic defenses in *Salicornia*. Their rapid response to oxidative signals and their interactions with each other reflect a sophisticated system designed for survival in saline environments. Future research should aim to unravel the regulatory crosstalk and synergistic actions among these molecules, which could offer valuable insights for developing crops with enhanced abiotic stress tolerance.

## 5. Maintenance of Osmotic Balance

A key consequence of salinity stress is its disruption of osmotic equilibrium, which impairs water uptake and alters cellular water potential [202,203]. This imbalance often results in dehydration, reduced turgor pressure, and subsequent inhibition of growth and metabolism [202]. To mitigate this, halophytes such as *Salicornia* employ a dual strategy: they accumulate inorganic ions and synthesize a wide range of organic osmolytes, including amino acids (AAs), glycine betaine (GB), soluble sugars, and sugar derivatives, for osmotic adjustment [203,204,205].

Glycine betaine, a quaternary ammonium compound, is well distributed among the plants of the family Amaranthaceae [77,194]. It is primarily derived via the pathway of two-step oxidation of choline, where choline monooxygenase catalyzes the oxidation of choline into betaine aldehyde dehydrogenase, which is further oxidized to yield GB by NAD^+^-dependent betaine aldehyde dehydrogenase [79,206]. Beyond being a typical osmoprotectant, GB prevents the photoinhibition caused by excess light energy, protects the Rubisco involved in CO_2_ fixation, induces the activity of ROS scavenging enzymes, and regulates the K^+^ efflux under salinity stress. Choline monooxygenase (CMO), a crucial enzyme which catalyzes the committing step in GB biosynthesis [79,207]. The *SeCMO* gene, encoding choline monooxygenase in *Salicornia europaea*, had been functionally evaluated by transfer into tobacco plants. The resulting transgenic tobacco demonstrated tolerance to 300 mM NaCl in MS medium, exhibiting GB concentrations nine times higher than the WT [208]. In another study, *SeCMO* promoter-β-glucuronidase chimeric gene containing five deletions enhanced the salt and drought stress tolerance in transgenic tobacco lines [77].

In addition to its role as a non-enzymatic antioxidant, proline functions as an organic osmolyte that enhances water uptake and facilitates water movement into cells, thereby contributing to osmotic adjustment under stress conditions [194,195]. In addition, it acts as a signaling molecule which assists in maintaining appropriate NADP^+^/NADPH ratios for cellular metabolic stability [194,195]. Proline is generally synthesized in plants via the glutamate pathway under stress conditions, which is composed of two crucial enzymatic catalyzers: delta-1-pyrroline-5-carboxylate synthetase (P5CS) and delta-pyrroline-5-carboxylate reductase (P5CR) [209,210,211]. Proline dehydrogenase and proline oxidase are involved in the catabolism of proline which takes place in mitochondria [50,196,210,211,212].

In *Salicornia persica* and *Salicornia europaea*, proline accumulation increases proportionally with salinity levels, indicating a salt-responsive regulation of proline metabolism [48,152,200,201,213]. Comparative studies between *Salicornia europaea* and *Suaeda maritima* revealed that both species respond to salinity by synthesizing multiple osmolytes, including GB and proline, suggesting a synergistic contribution to stress mitigation [213]. Additionally, the *SeProT* gene, which encodes a proline transporter in *Salicornia europaea*, was analyzed for its expression under different salinity conditions and growth stages [134]. Results showed that both high salinity and salt-free conditions significantly upregulated *SeProT* expression in shoots and roots, indicating that *Salicornia* perceives both salt excess and deficiency as stress conditions. This further underscores the involvement of proline in balancing osmotic stress across a wide range of environmental conditions [46].

Alkaloids are basic bioactive compounds, some of which have been reported to act as protective agents against oxidative stress and contribute to osmotic adjustment by accumulating under salinity stress in plants [193,214,215]. Metabolomic analysis of *Salicornia europaea* revealed that some alkaloids such as betanin and 3-O-acetylhamayne are significantly accumulated in root and shoot samples under increasing salinities. This suggests the involvement of alkaloids for the salinity tolerance of *Salicornia*, but further investigations are required to elucidate the relevant mechanisms [193].

In addition to amino acids, their derivatives, and alkaloids, soluble carbohydrates—including sugars and polyols—play a crucial role in maintaining osmotic balance [49,50,216]. A study involving the germination and growth of *Salicornia europaea* and *Salicornia persica* seeds in MS medium under varying salinity conditions showed increasing osmotic potential of cell sap with higher salt levels, as measured by vapor pressure osmometry [217]. Quantitative analysis revealed increased levels of reducing sugars, oligosaccharides, and soluble sugars, while polysaccharide content declined at higher salinity [217]. These results indicate that soluble carbohydrates contribute significantly to osmotic regulation, particularly under salt-induced stress.

Taken together, the synthesis and accumulation of organic osmolytes—such as GB, proline, alkaloids, and sugars—form a central component of *Salicornia*’s strategy for maintaining osmotic balance under saline conditions. While glycophytes also produce some organic solutes under salinity, *Salicornia* exhibits a more diverse and robust osmolyte profile. Moreover, unlike glycophytes, *Salicornia* can efficiently utilize both organic and inorganic osmolytes, particularly sodium ions (Na^+^), to maintain osmotic homeostasis. Since the use of inorganic solutes is energetically more efficient than synthesizing organic compounds, *Salicornia* prioritizes Na^+^ uptake as a primary osmotic adjustment strategy [218,219,220]. This dual capacity underscores the remarkable adaptability of *Salicornia* to thrive in saline environments.

## 6. Phytohormonal Regulation

Phytohormones are small chemical messengers that function as cellular signals, regulating a wide range of physiological processes, even at very low concentrations [221,222,223]. In addition to their roles in growth and development, they are key mediators of plant responses to both abiotic and biotic stresses, including salinity [222,223,224]. Among these, abscisic acid (ABA) is particularly responsive to abiotic stress, whereas ethylene and jasmonic acid (JA) are more prominently involved in biotic stress responses [222,224].

ABA, synthesized through the oxidative cleavage of carotenoid precursors, plays a central role in salt stress adaptation [221,225]. One of its most immediate actions under salinity is to induce stomatal closure, reducing water loss through transpiration and enhancing water-use efficiency [221,225,226]. Beyond this, ABA contributes to salinity tolerance by regulating ion homeostasis, activating ROS-scavenging mechanisms, stimulating osmolyte biosynthesis, and inducing salt-responsive gene expression [200,227]. For example, the *SbASR-1* gene cloned from *Salicornia brachiata* was expressed in transgenic tobacco and groundnut (*Arachis hypogaea*), resulting in enhanced tolerance to both salt and drought stresses. These transgenic lines exhibited improved seed germination, root length, leaf area, seedling biomass, chlorophyll content, membrane stability, and water retention compared to wild-type (WT) plants [200]. The downregulation of stress indicators such as electrolyte leakage and excessive proline accumulation further supported the reduced stress levels in the transgenic lines. Collectively, these results highlight *SbASR-1* as a promising gene for engineering salinity-tolerant crops.

Jasmonates (JA and its derivatives), biosynthesized from α-linolenic acid using cis-12-oxophytodienoic acid as the precursor, are involved in different physiological functions of plants, including plant growth, seed germination, senescence, and plant defense responses [228,229,230]. In addition to these roles, jasmonate signaling pathways have been shown to play a critical role in regulating salt stress responses in many plant species [231,232,233]. This suggests that JA may also contribute significantly to salinity tolerance in *Salicornia*. However, studies specifically investigating the role of jasmonic acid in *Salicornia* under salt stress are currently limited. Therefore, further research is needed to elucidate its function and underlying mechanisms in *Salicornia*’s salt stress adaptation. Gibberellins (GAs) are well known for regulating seed germination, cell elongation, and fruit development, but they also play an emerging role in salt stress tolerance [234,235]. In *Salicornia bigelovii*, the application of GAs during seed germination improved salt tolerance thresholds. Similarly, GAs alleviated salt-induced dormancy in *Salicornia rubra*, indicating its important function in overcoming germination barriers imposed by saline conditions.

The remarkable salinity adaptation of *Salicornia* is further supported by the action of ethylene, a key phytohormone that intricately modulates plant growth and abiotic stress responses [236,237,238]. Ethylene contributes to salinity tolerance by promoting ionic balance (Na^+^/K^+^), enhancing ROS scavenging through the induction of antioxidant systems, and facilitating the uptake of essential nutrients such as nitrates and sulfates [236,237]. Experimental evidence supports its role in *Salicornia* species: ethephon treatment has mitigated the inhibitory effects of salinity on seed germination in *Salicornia utahensis* [238], and has partially alleviated salt-induced dormancy in *Salicornia rubra* [239]. At the molecular level, ethylene regulates stress-responsive gene expression through interaction with APETALA2/ethylene response factor (AP2/ERF) transcription factors [234,235]. Among these, dehydration-responsive element binding (DREB) transcription factors—a key subgroup within the AP2/ERF family—have been linked to salinity tolerance in *Salicornia* [240,241]. These transcription factors are known to activate genes involved in osmotic adjustment, ion regulation, and oxidative stress mitigation, suggesting that ethylene signaling may play an important role in coordinating transcriptional responses under salt stress [240,241,242]. Collectively, these findings highlight the pivotal role of ethylene in modulating salinity tolerance in *Salicornia* through its integration with signaling pathways and gene regulatory networks.

Melatonin, a pleiotropic molecule recently recognized as a plant hormone, has shown promise in mitigating a variety of abiotic stresses, including salinity [243,244]. In *Salicornia fruticosa* grown in floating systems under media derived from prior *Salicornia* cultivation, foliar application of melatonin (0, 100, 200, and 400 µM) significantly enhanced plant growth and yield [245]. Notably, plants treated with 200 µM melatonin in peach leachate medium exhibited superior phytochemical profiles, including higher antioxidant activity, total phenolic content, and flavonoid accumulation [245]. Furthermore, combined foliar application of melatonin and ABA enhanced plant growth, morphological traits, and seed yield in *Salicornia europaea* under high-salinity conditions [36]. These findings suggest that melatonin could be an effective agent in improving *Salicornia* cultivation under saline environments. However, further research is needed to fully understand its signaling interactions and downstream targets.

Altogether, these studies illustrate that Salicornia employs a dynamic and multifaceted phytohormonal network to modulate stress responses. By integrating hormonal signals that regulate ROS detoxification, antioxidant production, osmotic balance, and gene expression, *Salicornia* maintains cellular function and resilience under harsh environmental conditions.

## 7. Signal Transducing Cascades

Salinity stress disrupts the ionic, osmotic, and oxidative balance in plants, necessitating efficient signal perception and transduction to activate adaptive responses at physiological, biochemical, and molecular levels [246,247]. Among the key components of plant signaling systems are protein kinases and phosphatases, which mediate the reversible phosphorylation of proteins—an essential regulatory mechanism in stress signal transduction and defense activation [247,248,249].

One of the most well-characterized signaling pathways in plants is the mitogen-activated protein kinase (MAPK) cascade, a conserved module in nearly all eukaryotes. This cascade typically involves a three-tiered phosphorylation sequence: a MAP kinase activating kinase (MAPKKK) activates a MAP kinase-activating kinase (MAPKK), which in turn activates a target MAP kinase (MAPK) [248,249]. Through this hierarchical structure, the MAPK cascade facilitates the transmission of extracellular stress signals to appropriate intracellular responses.

Functional studies in *Salicornia* have revealed important roles for MAPK signaling in salt stress adaptation. For instance, the *SeMAPKK* gene isolated from *Salicornia europaea* was introduced into *Arabidopsis thaliana*, resulting in enhanced salinity tolerance. Transgenic lines exhibited an increase in *SeMAPKK* expression from 2.99-fold at 0.25 M NaCl to 13.18-fold at 0.75 M NaCl, relative to wild-type (WT) plants [246]. However, expression declined to 1.28-fold at 1.0 M NaCl, suggesting a defined expression threshold under extreme salt conditions. Phenotypically, WT plants exposed to salinity displayed chlorosis and stunted growth, while the transgenic plants maintained healthier growth under the same conditions [246].

Similarly, in *Salicornia brachiata*, the expression of the *SbMAPKK* gene increased significantly under 0.1 to 0.5 M NaCl treatments but was downregulated at 1.0 M NaCl, mirroring the trend observed in *Salicornia europaea* [246,250]. Moreover, *SbMAPKK* expression was also strongly induced by dehydration and cold stress, indicating its broader involvement in multiple abiotic stress responses. Phylogenetic analysis classified *SbMAPKK* as an intronless gene belonging to group D of the MAPKK family, which is known for its stress-responsive regulatory roles [250].

These findings underscore the importance of MAPK cascades in salinity signaling, particularly the MAPKK module, in conferring salt tolerance in *Salicornia*. The observed expression dynamics suggest that these genes operate within an optimal activation range and are tightly regulated in response to salinity and other environmental cues. Moving forward, deeper investigation into the downstream targets of MAPK signaling, as well as its crosstalk with other hormonal and stress-related pathways, is essential for a comprehensive understanding of how *Salicornia* integrates signal transduction into its broader stress adaptation network.

The auxin signaling pathway plays a critical role in plant tolerance to salinity and other abiotic stresses [236,251,252,253]. As a key phytohormone, auxin regulates gene expression by acting as a chemical messenger through a family of transcription factors known as auxin response factors (ARFs) [252,253]. Under salinity stress, auxin also contributes to adaptive growth responses by promoting root architecture development and participating in cell wall remodeling processes [236,253]. Moreover, auxin exhibits crosstalk with other stress-responsive signaling pathways and compounds, forming an interconnected network that orchestrates complex stress responses [236,252,253]. In *Salicornia europaea*, salinity stress has been shown to significantly enhance the expression of auxin-responsive transcription factors and Aux/IAA family proteins in shoot tissues [254]. Among these, NAC family transcription factors appear to closely interact with auxin to modulate growth under stress conditions [254]. Altogether, these findings suggest that the auxin signaling network is integral to *Salicornia*’s salinity stress tolerance. Nevertheless, further research is needed to elucidate the underlying molecular mechanisms and the nature of their interactions with other regulatory pathways.

## 8. Transcriptional and Post Transcriptional Regulation

Transcriptional regulation plays a crucial role in the mechanisms underlying plant adaptation to abiotic stresses, including salinity [255,256,257,258]. Many transcription factors (TFs) that regulate the expressions of the genes related to the salt stress responses have been identified and characterized in *Salicornia* [241,242,259]. Among them, DREB TFs, members of the AP2/ERF family, have been found to play a key role in regulating genes responsive to multiple abiotic stresses, including drought, salinity, and cold in *Salicornia brachiata* and *Salicornia bigelovii* [240,241]. These transcription factors contribute to the broad activation of protective genes involved in osmotic adjustment, ion homeostasis, and the mitigation of oxidative damage, thereby explaining the multifaceted stress tolerance observed in these halophytes [260,261]. For instance, transgenic tobacco overexpressing a DREB TF cloned from *Salicornia brachiata* (*SbDREB2A*) exhibited higher K^+^/NA^+^ ratio, improved water content, water use efficiency, membrane stability, and chlorophyll content. Additionally, these transgenic plants showed reduced levels of ROS, MDA, H_2_O_2_, and electrolyte leakage compared to wild-type (WT) plants [240]. Moreover, expression of abiotic stress-responsive genes (*Hsp18*, *Hsp26*, and *Hsp70*), other TFs (AP2 domain-containing TF, HSF2, and ZFP), and signaling components (PLC3 and Ca^2+^/calmodulin) was upregulated under salt, heat, and drought stresses in the transgenic tobacco plants [241]. Similarly, nine AP2 TFs involved in osmotic dehydration were significantly upregulated at the early stage of salinity stress in *Salicornia europaea* [41]. Furthermore, *SbDREB2A* has imparted stress tolerance in recombinant *Escherichia coli*, where growth was enhanced in basal LB medium supplemented with NaCl, polyethylene glycol (PEG), and mannitol, separately [240].

In addition to their established role in transcriptional regulation, DREB TFs have also been implicated in modulating epigenetic mechanisms, such as DNA methylation, contributing to salinity tolerance in plants [242,262]. A study evaluating DNA methylation in transgenic tobacco lines overexpressing *SbDREB2A* (cloned from *Salicornia brachiata*) found that the most successful transgenic lines had higher methylation percentages and polymorphisms, suggesting that *SbDREB2A* modifies methylation processes to enhance salt stress tolerance. In contrast, no significant difference in demethylation was observed between transgenic and non-transgenic tobacco plants, indicating that this gene may specifically influence DNA methylation for stress alleviation [262]. These findings highlight the need for further investigation into the epigenetic mechanisms underlying *Salicornia*’s stress adaptation.

Among these transcription factors, MYB proteins—particularly the plant-specific R2R3-MYB family—are key regulators of abiotic stress responses [263,264,265,266]. They enhance salt tolerance by modulating networks of stress-responsive genes involved in osmotic adjustment, ion homeostasis, antioxidant defense, and the biosynthesis of protective barriers such as cutin, wax, and suberin [267,268,269]. In *Salicornia brachiata*, MYB transcription factors such as *SbMYB15* and *SbMYB44* have been shown to be strongly induced under salinity and contribute to stress tolerance by regulating downstream protective genes [259,267]. For example, they enhanced the salinity and dehydration tolerance in transgenic tobacco, demonstrating improved stomatal conductance, water use efficiency, photosynthesis rate, K^+^/Na^+^ ratio, and membrane stability. Additionally, transgenic plants exhibited reduced levels of H_2_O_2_ and $O_{2}^{•-}$ compared to WT plants under stress. Moreover, the transcription activity of transferred *SbMYB15* was exhibited by the enhanced expression of salt-stress-responsive genes of transgenic tobacco such as *SOD*, *CAT*, *LEA5*, *ERD10D*, *PLC3*, *LTP1*, *HSF2*, *ADC*, and *P5CS* under salinity stress conditions compared to WT [166]. Similarly, *SbMYB44* was responsive to salt, desiccation, high temperature, and cold stresses. Expression of this gene in transformed *Saccharomyces cerevisiae* exhibited higher growth rates under salinity and desiccation stress compared to non-transformed cells, further indicating the functional relevance of MYB TFs from *Salicornia* in abiotic stress tolerance [267].

In addition to MYB transcription factors, another salt- and drought-responsive gene, *SbSDR*, shows a regulatory function resembling a transcription factor. Although it belongs to the SDR enzyme family in *Salicornia brachiata*, it has served as a molecular switch in transgenic tobacco by regulating the expression of its large number of stress-responsive genes [201]. Transgenic tobacco overexpressing *SbSDR1* exhibited improved seed germination percentage under salt and osmotic stress. Subsequently, the developed transgenic seedlings exhibited higher relative water contents, membrane stability indexes, and proline and total soluble sugar contents, while producing less ROS (MDA and H_2_O_2_) compared to WT. These results underscore SbSDR1’s potential to coordinate multiple stress adaptation mechanisms [201].

The WRKY family, another key group of TFs, is also involved in plant responses to salinity, drought, and temperature extremes [270,271,272,273]. WRKY proteins regulate ion transport, antioxidant defense, osmotic balance, and hormonal signaling [271]. An in silico study identified two W-box WRKY binding sites in the promoter regions of the SOS1 gene in *Salicornia brachiata* and *Salicornia dolichostachya* [274]. While functional studies in *Salicornia* remain limited, WRKY TFs have been shown to play vital roles in salt stress tolerance in related halophytes such as *Suaeda australis* [275], suggesting a likely parallel role in *Salicornia*.

Similarly, NAC transcription factors are widely known for their involvement in abiotic stress tolerance [254,276,277]. Transcriptomic analysis of *Salicornia europaea* under salt stress identified three NAC family members that interact with auxin signaling pathways to regulate growth and stress mitigation. A comparative analysis between *Salicornia dolichostachya* and the glycophyte *Spinacia oleracea* revealed a 10-fold higher expression of the SOS1 gene in *Salicornia dolichostachya*, which was attributed to the presence of NAC binding sites in the SOS1 promoter [254]. These results point to the role of NAC TFs in mediating *Salicornia*’s exceptional stress responsiveness.

In summary, transcription factors play a central role in coordinating *Salicornia*’s response to salinity and other abiotic stresses. Through regulation of ion homeostasis, antioxidant activity, osmotic balance, and stress-responsive gene networks, TFs serve as master regulators. Given the frequent crosstalk between TFs and other components of stress response pathways, further studies are needed to fully elucidate their integrated roles and regulatory interactions.

In addition to transcriptional control, post-transcriptional regulation by microRNAs (miRNAs) and small interfering RNAs (siRNAs) also contributes to stress tolerance [35,278,279]. These small non-coding RNA molecules that regulate gene expressions play a significant role in plant adaptation to salinity stress by modulating the expression of stress-responsive genes [35,278,279]. A study identified 12 salt-responsive miRNAs and one siRNA from *Salicornia brachiata*, which were predicted to target 67 putative genes involved in stress responses. These include genes encoding salt-stress protein, heat-stress protein, cytochrome P450-like TATA box binding protein, mitochondrial glycol protein, polyubiquitin protein, serine/threonine protein phosphatase, CCAAT-box binding transcription factor, and zinc-binding protein. In *Salicornia europaea*, small RNA transcriptome analysis revealed that the expression of 43 conserved and 13 novel miRNAs was significantly altered in response to salt stress. The putative targeted unigenes of those miRNAs encode a wide range of crucial proteins including different TFs, highlighting their critical role in modulating gene networks essential for salinity tolerance of *Salicornia* [279].

## 9. Salt-Responsive Proteins

In addition to previously discussed adaptive mechanisms, several salt-responsive proteins have been identified as key contributors to the salinity tolerance observed in *Salicornia* [26,280,281]. These proteins participate in diverse processes such as stress signal transduction, ion homeostasis, antioxidant defense, and photosynthetic regulation, highlighting the multilayered nature of *Salicornia*’s stress resilience [26,280,281].

One such group of proteins is the universal stress proteins (USPs), which play a crucial role in various cellular responses to stress conditions and physiologically interact with plant growth and development, ion scavenging, and intracellular transport [282]. In *Salicornia brachiata*, the *SbUSP* gene encodes for a protein that belongs to the USP family, interacts with adenosine monophosphate, and contains relevant motifs for phosphorylation, ATP binding sites, and glycosylation. The transcript level of the *SbUSP* gene was upregulated by exposure to salt, drought, heat, and cold stress conditions, while maximum upregulation of 7.8-fold expression was observed by exposing *Salicornia brachiata* plants to 0.25 M NaCl treatment for 24 h. The expression of synthesized recombinant *SbUSP-GST* gene (designed by fusing *SbUSP* gene with *GST* gene) in *Escherichia coli* BL21 cells enhanced their salinity tolerance significantly compared to *Escherichia coli* BL21 cells expressing only the *GST* gene (control) and vector control (VC) cells [280]. These findings suggest that *SbUSP* enhances salinity tolerance by acting as a molecular switch in stress signaling mechanisms.

Another stress-responsive gene, *SbSRP*, a novel stress-related protein-encoding gene of *Salicornia brachiata*, localized in the plasma membrane has been characterized by over-expressing in tobacco under salt stress. This resulted in lower ROS content, enhanced relative water and chlorophyll contents and higher accumulation of proline, free AA, sugars, starch, and polyphenols, under salt and osmotic stress conditions in transgenic tobacco plants [280]. Furthermore, expression of *SbSRP* in transgenic tobacco triggered increased transcript level of several other stress-responsive genes, including an over 8-fold increase in *Nt-APX* under stress conditions compared to WT/VC plants. Additionally, antioxidant encoding genes (*Nt-CAT*, *Nt-SOD*) and TFs (*Nt-DREB* and *Nt-AP2*) were upregulated under stress conditions compared to WT/VC plants [280].

Similarly, overexpression of a novel gene cloned from *Salicornia brachiata*, the galactosyl transferase-like (*SbGalT*) gene, in transgenic tobacco plants improved seed germination, plant growth, antioxidant enzyme activity, and K^+^/Na^+^ ratio under salinity and osmotic stresses compared to WT and VC plants. In addition, enhanced stomatal activity, quantum yield, operating efficiency of PSII, electron transport, photochemical and non-photochemical quenching, and intercellular CO_2_ level resulted in higher photosynthetic efficiency even under stress conditions [281]. Therefore, *SbGalT* is a promising candidate for the bioengineering of crop plants to enhance salt stress tolerance.

Another important gene, *SbSI-1*, encodes a nuclear protein from *Salicornia brachiata* that conferred enhanced tolerance to both drought and salinity when expressed in transgenic tobacco [283]. These plants exhibited reduced ROS accumulation and oxidative damage, increased antioxidant activity, higher photosynthetic rates, improved membrane stability, and elevated polyphenol content, all of which contributed to greater stress resilience. Correspondingly, key antioxidant enzyme genes (*NtSOD*, *NtAPX*, *NtCAT*) and transcription factors (*NtDREB2*, *NtAP2*) were significantly upregulated under stress in the transgenic plants compared to WT [283].

These findings demonstrate that salt-stress-responsive proteins and their associated genes play integral roles in *Salicornia*’s abiotic stress tolerance. They illustrate the functional interconnectedness of biochemical, molecular, and physiological responses that collectively enable the plant to survive and thrive in high-salinity environments. These genes hold significant potential for the development of stress-resilient crops through genetic engineering and molecular breeding.

## 10. Crosstalk and Interactions Among Salinity Tolerance Mechanisms

Salinity, drought, and other abiotic stresses trigger multifaceted defense responses in plants, governed by complex interactions among signaling molecules, transcriptional networks, and physiological adaptations [284,285]. The tolerance observed in halophytes like *Salicornia* is not the result of isolated pathways but rather a coordinated network of signaling systems that function in an integrated manner to mitigate stress impacts [101,284,285].

Central to this regulatory network are key signaling components—including calcium ions (Ca^2+^), reactive oxygen species (ROS), nitric oxide (NO), hydrogen sulfide (H_2_S), hydrogen peroxide (H_2_O_2_), mitogen-activated protein kinases (MAPKs), and phytohormones—which collectively orchestrate responses to salinity at both cellular and systemic levels [285]. These components do not act in isolation; rather, they engage in dynamic crosstalk, amplifying or modulating each other’s signals to optimize plant stress responses.

Salt stress is first sensed by specialized receptors and ion channels in root cells, leading to a rapid influx of Ca^2+^ [102,286]. This rise in cytosolic Ca^2+^ acts as a pivotal second messenger, initiating downstream cascades such as the activation of calmodulin and other Ca^2+^-binding proteins. These, in turn, regulate a wide array of stress-responsive genes involved in osmoprotectant biosynthesis, ROS detoxification, and polyamine and nitric oxide signaling [101,102]. Notably, Ca^2+^ signaling is also essential for activating the SOS pathway, which plays a critical role in maintaining ion homeostasis under salt stress—particularly well-documented in *Salicornia* species [76,86,88,89].

Interlinked with Ca^2+^ signaling is the MAPK cascade, another conserved pathway that responds to salinity by relaying extracellular signals to stress-responsive gene expression. MAPKs can be activated through ROS and Ca^2+^ inputs, thereby serving as a convergence point for multiple stress signals and enabling fine-tuned transcriptional regulation [287]. In *Salicornia europaea*, the *SeCAX3* gene—encoding a putative Ca^2+^/H^+^ antiporter—was significantly upregulated in shoots under various stress stimuli, including Na^+^, Ca^2+^, Li^+^, ABA, and cold. Functional expression of *SeCAX3* in *Saccharomyces cerevisiae* enhanced tolerance to salt, drought, and cold stresses, underscoring its versatile role in abiotic stress adaptation [45].

Further supporting the importance of Ca^2+^ signaling, transcriptomic analyses in *Salicornia* have revealed the early induction of calcium-binding and calmodulin-related genes following salt exposure [28]. This indicates that the Ca^2+^-mediated signaling response is among the first lines of defense activated during salt stress, facilitating rapid cellular adjustment.

Adding another layer of complexity is nitric oxide (NO), a gaseous signaling molecule that interacts with both Ca^2+^ and ROS pathways. At optimal concentrations, NO plays a multifaceted role in modulating plant growth and stress responses [22,139,144]. In *Salicornia persica*, exogenous application of NO (via sodium nitroprusside) significantly improved growth and reproductive success under salinity stress. This was associated with enhanced activity of antioxidant enzymes, increased proline accumulation, and elevated chlorophyll content [139], highlighting NO’s central role in coordinating physiological resilience to salt stress.

These interconnected signaling networks exemplify the complexity and efficiency of *Salicornia*’s stress tolerance mechanisms. The integration of Ca^2+^, MAPKs, NO, and other signaling elements allows the plant to mount a rapid, robust, and finely regulated response to environmental challenges.

## 11. Cross-Tolerance of *Salicornia* to Abiotic Stresses

*Salicornia* species are widely recognized for their exceptional tolerance to salinity [288,289,290,291,292]. However, mounting evidence suggests that they also possess remarkable resilience to other abiotic stresses, including drought, heat, cold, and hypoxia [27,160,240]. This capacity for multiple stress tolerance is likely an evolutionary adaptation to the harsh and dynamic environmental conditions typical of their native saline and coastal habitats. Such environments often expose plants to simultaneous or sequential stresses, promoting the selection of common and overlapping adaptive mechanisms [293].

Although the physiological effects of different abiotic stresses vary, there are notable similarities—particularly between salinity and drought, both of which initially cause cellular dehydration through osmotic stress and turgor loss [294]. Cold stress, on the other hand, primarily exerts mechanical constraints on membrane fluidity and stability [294]. Despite these differences, plants often activate shared signaling pathways and stress-responsive genes, forming the basis for cross-tolerance strategies.

Experimental studies in *Salicornia brachiata* support the presence of such interconnected responses. For instance, the ascorbate peroxidase gene *SbAPX* showed enhanced transcript levels under salt, cold, abscisic acid (ABA), and salicylic acid (SA) treatments. When transferred into tobacco, *SbAPX* conferred improved tolerance to both drought and salinity, validating its role in overlapping stress-response pathways [160].

Similarly, *SbSDR1*, a transcription factor-like gene from *Salicornia brachiata*, has been functionally validated in transgenic tobacco, in which it alleviated osmotic, drought, and salt stress. The improved performance under multiple stress conditions suggests that *SbSDR1* regulates a suite of common adaptive responses, likely acting as a molecular switch [201].

Another key transcription factor, *SbDREB2A*, from *Salicornia europaea*, has demonstrated strong cross-tolerance potential. In transgenic tobacco, *SbDREB2A* conferred enhanced resistance to both hyperionic (salt) and hyperosmotic (drought) stresses, along with upregulation of heat shock protein genes [240]. Furthermore, recombinant *Escherichia coli* expressing *SbDREB2A* showed significantly increased survival under salt, drought, and heat stresses, highlighting the gene’s functional versatility [240].

While many studies assess individual stress responses [63,122,152,196], understanding plant adaptation under combined stress conditions is more ecologically relevant and critical for translational research. In this context, a study investigating the combined effects of salinity and tidal submergence in *Salicornia europaea* revealed that periodic tidal flooding with saline water (0.5 M NaCl) did not hinder plant growth. However, prolonged submergence reduced growth without causing mortality. Interestingly, expression analysis of hypoxia-responsive genes in *Salicornia brachiata* indicated that the species does not experience significant hypoxic stress during tidal flooding, suggesting a high level of physiological adaptation to such dynamic environments [295].

These findings emphasize the importance of evaluating multi-stress interactions to uncover the interlinked regulatory mechanisms that enable cross-tolerance. The broad-spectrum stress resilience observed in *Salicornia* not only highlights its ecological success but also positions it as a valuable genetic resource for improving abiotic stress tolerance in crop species.

## 12. Conclusions and Perspectives

Salinization has become a global concern, significantly impacting agricultural productivity and food security, and this challenge is projected to intensify in the coming decades. Improvement of in the salinity tolerance of crop plants can be considered one of the most effective ways to address this issue. *Salicornia* has the potential to luxuriantly grow in saline environments. Besides higher salinity tolerance, *Salicornia* is considered as an important cash crop due to its diverse applications in the food, feed, cosmetics, bioenergy, and pharmaceutical industries.

**Figure 4:**
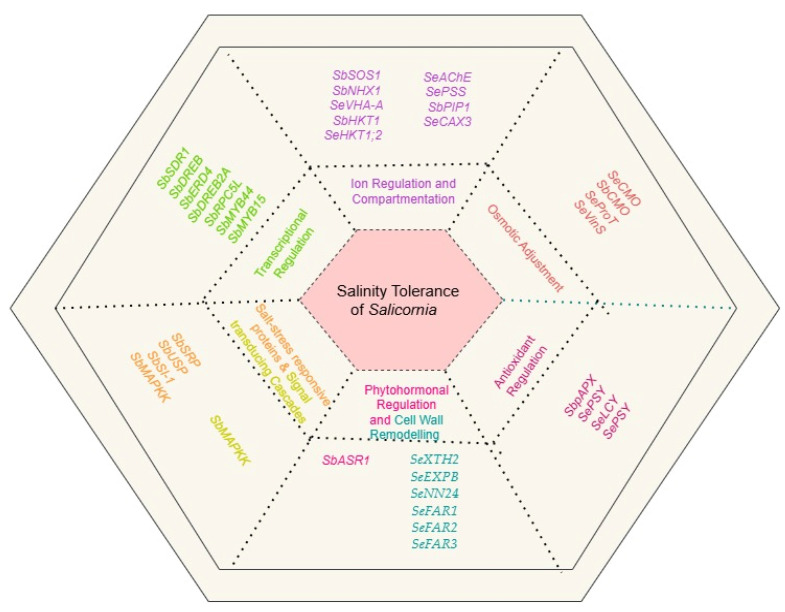
Salt-stress-responsive genes of *Salicornia* and categories of their major mechanisms. Six categories have been recognized, namely, genes involved in ion regulation and compartmentation, osmotic adjustment, antioxidant regulation, phytohormonal regulation, salt-stress-responsive proteins and signal transducing cascades, and transcriptional regulation (transcription factors). *Sb*: *Salicornia brachiata* except *SbHKT1*, which is cloned from *Salicornia bigelovii*; *Se*: *Salicornia europaea*. Different colored letters represent distinct salinity tolerance mechanisms.

*Salicornia*’s salt tolerance involves a complex interplay of physiological traits, biochemical mechanisms, metabolic pathways, and molecular networks. Elucidation of those mechanisms will enlighten the understanding of salt stress tolerance and provide insights into the development of salt tolerance of crop plants. This review has summarized the salinity tolerance at the gene, transcript, protein, and metabolite levels to provide a comprehensive understanding of salt stress tolerance mechanisms that would be important for the utilization of *Salicornia* as crop plants and as genetic resources for bioengineering applications [Figure 4, Table 1].

To date, several candidate genes involved in salinity tolerance have been isolated and functionally characterized. However, further research should be undertaken to study unrevealed aspects of salinity tolerance in *Salicornia*. For instance, chloride channel family genes are also considered crucial in plant salt stress responses, but there are scarce studies on the activity of chloride channel family genes in *Salicornia*. Phytohormones play a crucial role in mitigating salt stress in *Salicornia*, and few of them have been studied. However, novel hormones such as brassinosteroids, which has proven their capacity to enhance salinity tolerance in other halophytes, should be incorporated for the future investigations on *Salicornia*’s salt tolerance.

Reactive nitrogen species are produced under abiotic stresses and lead to nitrosative stress. However, it is largely unknown how they are regulated under salinity stress in *Salicornia*. Since salinity tolerance is a multigenic inherent trait, it may highly variable among species and subspecies levels. This emphasizes the requirement of breeding programs, as well as expanding future studies on salinity tolerance mechanisms to untouched *Salicornia* species. Although initial steps of molecular breeding of crop plants for salinity tolerance using selected *Salicornia* genes have been reported in several studies, almost all of them are limited to in vitro or control environment agriculture facilities. Therefore, it is important to extend such future studies up to real field applications.

Furthermore, most existing studies focus on individual stress factors. However, in natural environments, plants are often subjected to multiple concurrent stresses, such as salinity combined with drought or submergence. Investigating *Salicornia*’s responses to combined stress conditions is therefore essential to gain insights into its full adaptive potential. Currently, research in this area remains limited.

Another vital area of future research is the intricate crosstalk between signaling pathways and stress-responsive compounds. These interactions enable plants to integrate multiple signals and mount a coordinated response to diverse environmental stresses. Understanding such crosstalk in *Salicornia* could uncover novel regulatory networks that contribute to its exceptional tolerance, yet this area remains largely unexplored.

Lastly, while several studies have addressed *Salicornia*’s salinity tolerance at the genomic, transcriptomic, proteomic, and epigenetic levels, most of them rely on single-omics approaches. Integrating multi-omics strategies will yield more robust insights and help to elucidate how different molecular layers work together to support *Salicornia*’s adaptation to saline environments.

## Figures and Tables

**Table 1:** A list of genes in *Salicornia* species and their probable functions in imparting salt tolerance.

Salicornia Species	Gene	Function	Source
*Salicornia brachiata*	*SbGSTU*	Reduces secondary noxious by-products generated during oxidative stress and exhibited potential signaling functions	[142,162]
*Salicornia brachiata* *Salicornia europaea*	*SbNHX1* *SeNHX1*	Maintains ion homeostasis by regulating the sequestration of Na^+^ into vacuoles	[90,91]
*Salicornia bigelovi*	*SbHKT1*	Assists in maintaining K^+^/Na^+^ homeostasis by increasing the capacity of K^+^ uptake	[95]
*Salicornia europaea*	*SeHKT1;2*	Reduces Na^+^ retrieval from the xylem and enhances Na^+^ transport into shoot tissues	[96]
*Salicornia brachiata*	*SbMAPKK*	Phosphorylates proteins and other cellular substrates to regulate them over abiotic stress	[250]
*Salicornia brachiata*	*SbDREB2A*	Serves as a transcription factor (TF) by regulating the expression of stress- responsive genes	[240,241]
*Salicornia brachiata*	*SbMT-2*	Modulates the ROS scavenging and confers abiotic stress tolerance tolerance to plants	[23]
*Salicornia brachiata*	*SbSLSP*	Enhances ROS scavenging, efficiency of transporters and the stability of cell membrane, and improves clathrin-coated vesicle-mediated endocytosis, leading to efficient the stress signaling	[24]
*Salicornia brachiata*	*SbpAPX*	Involved in scavenging ROS and protecting cells against their toxic effects under salt and drought stress conditions	[160]
*Salicornia brachiata*	*SbSOS1*	Encodes a Na^+^/H^+^ antiporter located in plasma membrane that plays an important role in imparting salt stress tolerance to plants	[88]
*Salicornia brachiata*	*SbASR1*	Encodes stress-responsive nuclear protein functioning as a transcription factor which regulates expression of stress responsive genes	[200]
*Salicornia brachiata*	*SbUSP*	Encodes a membrane-bound cytosolic protein, regulates ROS accumulation, and is involved in maintaining ion homeostasis	[26]
*Salicornia brachiata*	*SbSRP*	Encodes transporter protein to transmit the environmental stimuli downward through the plasma membrane improving the abiotic stress tolerance	[280]
*Salicornia brachiata*	*SbSI-1*	Encodes a salt-responsive nuclear protein which enhances the antioxidant activity and maintains osmotic homeostasis	[283]
*Salicornia brachiata*	*SbRPC5L*	Regulates expression of many stress-responsive genes and transcription factors	[25]
*Salicornia brachiata*	*SbGalT*	Minimizes the buildup of reactive oxygen species (ROS) and maintains the membrane integrity	[281]
*Salicornia brachiata*	*SbSDR1*	Functions as a molecular switch and contributes to salt and osmotic tolerance	[201]
*Salicornia brachiata*	*SbERD4*	Encodes a plasma-membrane-bound protein which alleviates osmotic and salt stresses by moderating physio-biochemical processes	[296]
*Salicornia brachiata*	*SbMYB44* *SbMYB15*	Act as transcription factors which regulate a range of genes crucil for abiotic stress tolerance	[259,267]
*Salicornia europaea*	*SeXTH*	Encodes a cell wall manipulating enzyme, which improves cellular anatomy and physiology to mitigate abiotic stresses	[47]
*Salicornia europaea*	*SeXTH2*	Involved in cell wall remodelling by producing enzyme under abiotic stress	[64]
*Salicornia europaea*	*SeEXPB*	Encodes an expansin protein, which assists in cell wall remodelling via enhancing the expansion properties	[64]
*Salicornia europaea*	*SeNN24*	Encodes a TPL-like protein which is involved in cell wall modifications to alleviate salt stress	[68,69]
*Salicornia europaea*	*SeFAR1 SeFAR2 SeFAR3*	Involved in cuticular wax biosynthesis to enhance defense gainst abiotic and biotic stresses	[72]
*Salicornia europaea*	*SeCAX3*	Encodes a putative Ca^2+^/H^+^ antiporter which modulate ionic homeostasis under salt stress	[45]
*Salicornia europaea*	*SeAChE*	Believed to be involved in ion transport through channels by a similar way in animal systems	[100]
*Salicornia europaea*	*SeVHA-A*	Regulates the proton pumping reaction by stimulating the hydrolysis of PPi to energize the antiporters	[44]
*Salicornia europaea*	*SePSY*	Involved in carotenoid biosynthesis, which detoxifies ROS effectively	[63]
*Salicornia europaea*	*SeLCY*	Regulates the carotenoid biosynthesis and improve ROS scavenging potential	[297]
*Salicornia europaea*	*SeVinS*	Encodes a vinorine synthase, which is crucial for alkaloid biosynthesis to maintain osmotic balance	[46]
*Salicornia europaea*	*SeProT*	Regulates proline accumulation in response to salinity stress by encoding a proline transporter	[46]
*Salicornia bigelovii*	*SbPIP*	Serves as an aquaporin in plants which facilitates the water and ion transportation	[298]
*Salicornia herbacea*	*ShTIP*	Modulates a type of aquaporins in vacuoles required for ionic and osmotic stress adaptation	[299]

## Data Availability

No datasets were generated or analyzed during this study.
